# Interleukin-17 in Antifungal Immunity

**DOI:** 10.3390/pathogens8020054

**Published:** 2019-04-22

**Authors:** Florian Sparber, Salomé LeibundGut-Landmann

**Affiliations:** Section of Immunology, Vetsuisse Faculty, University of Zürich, Winterthurerstrasse 266a, CH-8057 Zürich, Switzerland

**Keywords:** Interleukin-17, fungal pathogens, fungal commensals, *C. albicans*, host protection, immunopathology

## Abstract

The field of IL-17 biology has received much attention over the last decade owing to the pathogenic role of this cytokine in psoriasis and other autoinflammatory disorders and the successful implementation of IL-17-targeting therapies in patients suffering from these diseases. IL-17-mediated pathologies are contrasted by the important host beneficial effects of this cytokine. IL-17 is essential for regulating microbial colonization in barrier tissues. Rare congenital defects in the IL-17 pathway exemplify the relevance of IL-17 in protective immunity against the opportunistic fungal pathogen *C. albicans*. However, more recently, evidence is accumulating that IL-17 can also provide protection against fungi other than *C. albicans*. Importantly, protective IL-17 responses directed against commensal fungi can, under certain conditions, promote inflammation with detrimental consequences for the host, thereby assigning fungi a new role as disease-promoting factors apart from their role as potential infectious agents.

## 1. IL-17 Immunity in Health and Disease 

Interleukin (IL)-17 is an ancient and therefore well-conserved cytokine, although it was discovered rather late compared to other key cytokines [1]. Since its first description, and in particular over the last 15 years, IL-17 has been intensely studied in the context of human and mouse immunology as well as in very distant organisms such as the pacific oyster, sea urchin or sea squirt [2,3,4]. The IL-17 cytokine family consists of six members (IL-17A through IL-17F), among which IL-17A and IL-17F are the best characterized cytokines [5]. This is attributed to their high sequence homology and close functional relationship. In this review, we focus on IL-17A and IL-17F and use the term IL-17 interchangeably for both cytokines.

IL-17 has gained much attention for its implication in autoinflammatory disorders. Dysregulation of the IL-17 pathway is associated with the development of diseases such as psoriasis and rheumatoid arthritis. Three therapeutic antibodies targeting IL-17A or its receptor and another three antibodies targeting one of the two subunits of the IL-17-regulating factor IL-23 have been brought to market over the past four years [6]. These antibodies demonstrate surprisingly rapid efficacy against moderate to severe forms of plaque psoriasis with short treatment periods. In addition, recent trials suggest that patients suffering from other diseases such as ankylosing spondylitis and psoriatic arthritis would benefit from IL-17 blockade [7]. 

Importantly however, IL-17 confers not only detrimental effects to the host, but this cytokine is also critical for maintaining an equilibrium between the host and the microbiota in barrier tissues during homeostasis. In the gut, IL-17 is produced by a subset of CD4^+^ T cells, called Th17 cells, as well as by innate lymphoid cells in response to commensal microbes and in turn prevents microbial dysbiosis by a regulating effector mechanism in the epithelium that prevents the overgrowth of microbes. As a consequence, the use of anti-IL-17 therapy may come at the price of disrupting microbial homeostasis, potentially resulting in opportunistic infections. This is exemplified by the adverse effect of anti-IL-17 and IL-17R treatment for patients with Crohn’s disease [8,9]. These results are backed up with studies in mice documenting a protective role of IL-17 in controlling the gut microbiota [10,11]. Of note, in contrast to biological therapies targeting IL-17, those targeting IL-23 showed beneficial effects in patients suffering from intestinal inflammatory disease, indicating that some actions of IL-23 may be independent of IL-17 immunity. 

One of the very few adverse effects of IL-17-targeting therapies in psoriasis patients described so far has been the occurrence of fungal infections, particularly superficial infections caused by *Candida*, in a small proportion of the treated patients [12]. This does not come as a surprise given the essential role of IL-17 in protection from this fungus, which is now well accepted based on patient and mouse studies. 

## 2. IL-17 Immunity against *C. albicans*

Rare genetic defects in the IL-17 pathway indicated a non-redundant role of IL-17 in protective immunity against fungal infections, especially those caused by *Candida albicans* and rarely non-albicans species of *Candida*. Early on, it was observed that patients suffering from chronic mucocutaneous candidiasis (CMC) exhibit reduced production of IL-17 and IL-22 cytokines [13]. Later on, rare genetic defects were identified in patients suffering from familial forms of CMC with symptoms manifesting in the oral mucosa, the skin and the nails [14]. Mutations were identified in a small number of selected genes, in particular those encoding for IL-17F, the IL-17 receptor subunits A and C, the signaling adaptor ACT1 and the IL-17-instructing transcription factor RORγt [15,16,17,18]. By far, the most common mutations associated with CMC are STAT1 GOF (gain of function) and STAT3 LOF (loss of function) alleles [19,20,21,22,23,24]. In addition, mutations in the genes coding for DOCK8 [25,26] and for the adaptor molecule CARD9, which links innate fungal recognition to Th17 induction are also associated with increased susceptibility to CMC [27,28,29,30,31]. CARD9 is the only gene also associated with invasive forms of fungal diseases, including deep-seated dermatophytosis, fungal encephalitis and extrapulmonary aspergillosis, indicating that this factor regulates functions in antifungal immunity beyond the epithelial barrier [32]. In the cases of CNS candidiasis and extrapulmonary aspergillosis, the defect in CARD9 was mapped to the lack of neutrophil recruitment [28,33]. 

Of all forms of superficial candidiasis, vulvovaginal candidiasis (VVC) is the most common disease, affecting not only immunocompromised but also otherwise healthy women [34]. One of the most prominent predisposing factors for VVC is dysbiosis induced by antibiotics [35]. Primary immunodeficiency patients with defects in the IL-17 pathway frequently display symptoms of VVC in combination with those affecting the oropharynx, the skin and/or the nails, indicating a role of IL-17 in the prevention of this disease. However, in contrast to mucocutaneous candidiasis, the exact role of IL-17 in VVC remains controversial [36,37]. The immunopathogenesis of VVC is now believed to be rather a consequence of neutrophil dysfunction due to heparin-sulfate-mediated inhibition [38].

The relevance of IL-17-mediated antifungal defense in the oro-gastrointestinal tract and the skin was investigated in detail using experimental models of superficial *Candida* infection in mice. Studies employing the models of oropharyngeal candidiasis (OPC) or epicutaneous candidiasis greatly improved our current knowledge about the cellular and molecular mechanisms underlying IL-17-mediated immunity against *Candida* in barrier tissues.

## 3. Mechanisms of IL-17 Induction and Function

As a consequence of *C. albicans* being a commensal in humans, *Candida*-specific memory cell populations can be found in all human individuals, which are preferentially polarized towards the Th17 subset [39,40,41]. Likewise, a selective type 17 immune response is triggered in mice that are experimentally infected with *C. albicans* in the oral cavity without concurrent induction of IFN-γ or IL-4/-5/-13 [42,43]. The antifungal Th17 response in mice also generates memory [42] that augments antifungal protection upon re-infection via the same route [44,45]. Of note, the *C. albicans*-specific Th17 response is highly conserved and independent of genetic variations, which exist within the species of *C. albicans* and which entail differences in the virulence between individual fungal isolates [46,47]. Using low-virulent strains of *C. albicans* in experimental OPC has allowed the establishment of a model of persistent infection that closely mimics the situation of fungal commensalism in humans [46,47]. 

However, the original and still most commonly used mouse models of superficial candidiasis employ the highly virulent strain SC5314 or derivatives thereof. This strain triggers a very rapid inflammatory response in mice that are not usually colonized with *C. albicans* under specific pathogen-free conditions. The acute and very strong activation of the immune system in response to primary exposure to the fungus results in rapid clearance of the fungus within only a few days [43,48].

The natural resistance of mice to *C. albicans* is strictly dependent on a functional IL-17 pathway [43,48]. The rapid infection kinetics with strain SC5314 preclude a contribution of Th17 to fungal control. It was thus suggested that innate immune cells participate in the production of IL-17. Indeed, γδ T cells, innate lymphoid cells (ILCs) and a population of αβ T cells (also called “natural Th17 cells” by some) have been identified in the oral mucosa as an important source of IL-17 shortly after the onset of experimental OPC [49,50]. Of these three subsets, the identity of the αβ T cells remains the most poorly characterized, and their putative relationship to tissue-resident memory cells remains to be clarified. The three IL-17-producing innate cell populations act in a complementary and partially redundant manner in the oral mucosa to ensure rapid production of IL-17 at the onset of infection for rapid fungal control. The lack of one or two of these populations, as is the case in TCRγδ- or RAG-deficient animals, results in a less severe phenotype than those observed in the absence of the entire lymphoid compartment (e.g., in *Rag-/- Il2rg-/-* mice) or the full IL-17 signaling pathway (e.g., in *Il17ra-/-* mice) [48]. In the skin, dermal γδ T cells rather than αβ T cells or ILCs are the main producers of innate IL-17 in response to experimental infection with *C. albicans* [51], consistent with the numerical and functional dominance of γδ T cells in this organ. Neutrophils were shown to serve as a source of IL-17 in the context of ocular mold infections [52]. However, no evidence exists that they contribute to IL-17 production during oropharyngeal or epicutaneous candidiasis. The role of ILCs and innate lymphocytes in antifungal defense in humans who are constantly exposed to *C. albicans* has not been assessed so far, but it can be speculated that they also contribute to protection in barrier tissues. 

IL-17 production by innate and adaptive immunity is instructed by polarizing cytokines, in particular IL-23, IL-1 and IL-6. Production of these cytokines in the infected tissue has been studied in depth during murine oropharyngeal and epicutaneous candidiasis with strain SC5314. A lack of any of these factors (or combinations of these factors) impairs IL-17 induction and protective antifungal immunity [43,45,48,49,50]. In the oral mucosa, tissue-resident Langerin^+^ dendritic cells (DCs) occupy a unique position as coordinators of the acute IL-17 response by co-producing all three IL-17-inducing factors in response to *C. albicans* [50]. In addition, IL-1 is also provided by the epithelium in response to the fungal peptide toxin candidalysin [53], which is strongly expressed during filamentation of virulent strains of *C. albicans*. Candidalysin causes damage in host cells and consequently facilitatesthe release of alarmins including IL-1α from the damaged cells [54].

Avirulent strains of *C. albicans* have been shown to induce an attenuated and delayed host response in experimentally infected mice [46,47], while the adaptive immune response is activated regularly and indistinguishably in response to both, high- and avirulent strains. This indicates that the fungal determinants of pathogenicity that induce a rapid inflammatory response to high-virulent strains are distinct from more conserved molecular patterns that instruct Th17 immunity for the surveillance of persistently colonizing fungus [46]. The mechanism of Th17 cell priming in response to avirulent strains of *C. albicans*, which induce only limited levels of the polarizing cytokines IL-1, IL-6 and IL-23 [46,47], remains to be determined.

The type of DCs driving innate vs. adaptive antifungal IL-17 immunity differ between different sites of infection. While Langerhans cells play a critical role at the onset of infection in the context of oropharyngeal candidiasis [50], they are redundant for the activation of adaptive Th17 immunity in this model, where infiltrating myeloid cells adopt the role of antigen-presenting cells [55]. In the skin however, Langerhans cells contribute to the priming of the adaptive antifungal response to *C. albicans* by providing IL-6, but instead they are not required for innate immunity in this tissue [56]. Importantly, research from the epicutaneous candidiasis model in mice has linked neuronal sensitization to antifungal immunity by demonstrating that TRPV1^+^ neurons in the skin sense *C. albicans* and instruct dermal CD301b^+^ DCs to produce IL-23, which in turn drives IL-17 production by dermal γδ T cells [51]. This is consistent with a growing body of literature on the regulation of immunological processes by peripheral neurons in homeostasis and disease [57]. Resident commensal microbiota have also been shown to regulate local IL-17 production and prevent fungal infection. *Corynebacterium mastidis*, a component of the microbiota of the ocular surface in mice and humans, stimulates tissue-resident γδ T cells to produce IL-17, which in turn is necessary to confer protection against invasive *C. albicans* infection of the eye [58].

While research on IL-17 in recent years has mainly focused on its cellular source, we still know relatively little about the host-protective effector functions of this cytokine contributing to fungal control. Neutrophils have been linked to type-17 immunity and IL-17 target genes are associated with neutrophil trafficking [59,60]. Indeed, neutrophils have a key role for protective antifungal immunity in the oral mucosa as they prevent dissemination of virulent *C. albicans* strains [61,62]. However, abrogation of IL-17 signaling during OPC does not affect neutrophil recruitment to the site of infection, nor does it impair the neutrophil antifungal activity [62]. Moreover, IL-17 induction during OPC with avirulent *C. albicans* strains is not sufficient for neutrophil recruitment into the infected tissue [46]. This indicates that neutrophils take action independently of IL-17, or even act upstream of local IL-17 production by secreting IL-17-instructing cytokines during the acute phase of OPC [50].

IL-17 exerts its effector function primarily by targeting non-hematopoietic cells in barrier tissue including epithelial cells and fibroblasts [63]. Indeed, deletion of the IL-17RA subunit from keratin 13-expressing oral epithelial cells phenocopied the susceptibility of IL-17RA full knockouts to OPC [64]. IL-17 induces the production of antimicrobial peptides (AMPs) by those cells, including S100A8 and S100A9 (together forming the heterodimeric complex calprotectin), lipocalin 2 and β-defensins [43]. These compounds have been shown to display direct fungicidal activity by permeabilizing the fungal cell wall [65] or by dampening fungal growth via sequestration of essential metal ions [65,66,67,68]. In addition to their antimicrobial activity, AMPs can also act as chemoattractants. In contrast to calprotectin and lipocalin 2, which are also highly expressed by activated neutrophils, the expression of β-defensins is largely restricted to epithelial cells [69]. β-defensin-3-deficiency was linked to impaired fungal control during murine OPC [64], while β-defensin-1 was reported to modulate host immunity with impaired neutrophil recruitment and reduced expression of inflammatory cytokines including IL-17, and consequently increased susceptibility to infection in the absence of β-defensin-1 [70]. The defect of AMP-deficient mice in antifungal immunity is not as rigorous as in mice lacking the entire IL-17 pathway, suggesting a certain degree of redundancy between different AMPs. In line with this, lipocalin 2, which is one of the most strongly upregulated factors in response to IL-17 induction in the oral mucosa, is dispensable for protective immunity [71] and the phenotype of β-defensin-3-deficient mice was not reproducible in our studies (Kirchner, Sparber and LeibundGut-Landmann, unpublished). Thus, the identification of the relevant effector molecules mediating IL-17-dependent immunity to *C. albicans* infection awaits further investigation.

## 4. IL-17 in Antifungal Immunity beyond *Candida*

The vast majority of all studies on the protective effects of IL-17 immunity against fungal infections has been focused on *C. albicans*. However, the mammalian host is exposed to diverse other fungi, including airborne fungi that we inhale and fungi to which our skin is exposed. Studies in mice have revealed a potential role of IL-17 in the defense against many of these fungi. Protection from *Pneumocystis carinii* [72] and the dimorphic fungi *Histoplasma capsulatum* [73,74] and *Blastomyces dermatitis* [75] has been shown to depend on a functional IL-17 pathway. Reminiscent of what is known from the IL-17 response to *C. albicans*, experimental infection of mice with *B. dermatitis* induces an acute response characterized by rapid production of IL-17 and GM-CSF by tissue-resident γδ T cells via a mechanism that involves IL-1α secretion from lung epithelial cells [76]. One of the most clinically relevant airborne fungal pathogens is *Aspergillus fumigatus*. In an experimental aspergillosis model, fungal killing was shown to depend on Dectin-1-mediated induction of IL-17 [77]. The relevance of IL-17 in lung immunity against *A. fumigatus* gained support from studies reporting an increased frequency of Th17 cells in the lung tissue of COPD and cystic fibrosis patients, who frequently suffer from pulmonary aspergillosis [78,79]. However, whether the fungus-specific Th17 cells in these patients mediate fungal control or, alternatively, may contribute to lung pathology that is associated with COPD remains an open question. Dysregulated Th17 responses have recently been linked to the induction of immunopathology and were associated with disease severity in asthma [80,81]. 

IL-17 immunity has also gained attention for providing protection from fungi on the skin. Various fungi beyond *Candida* can cause different forms of dermatomycosis. Dermatophytes are the most frequent skin fungal pathogens encountered in human and veterinary dermatology [82,83]. Early observations in human patients suggested a protective role of IL-17 against this class of fungi [84,85,86]. More recently, two independent studies in mice further underlined the crucial role of IL-17 for antifungal immunity against dermatophytes. Experimental infection of the skin with *Microsporum canis* resulted in the induction of a fungus-specific Th17 response in the skin draining lymph nodes. Analyzing the host response to dermatophytes in IL-17-deficient animals revealed that IL-17 not only limits dermatophyte growth on the skin, but also antagonizes a non-protective Th1 response against the fungus, which in the absence of IL-17 resulted in excessive skin inflammation and fungal overgrowth [87]. A similar protective role for Th17 has been demonstrated against the dermatophyte *Trichophyton benhamiae* [88]. While this most recent study confirmed the critical role of Th17 cells in protection from dermatophytosis, it reported a synergistic instead of an antagonistic role of IL-17- and IFN-γ-producing T cells to facilitate optimal fungal clearance [88]. The discrepancy between the two studies may be explained by differences in the two fungal genera, the mouse background and microbial status and/or details in the experimental protocol including the preparation of the mice for infection. However, the common feature remains the host protective role of IL-17 against dermatophytes. 

The most prominent fungal commensal organisms colonizing the skin are those of the genus *Malassezia* [89]. Besides their role as commensals with potential benefits for the host during homeostasis, *Malassezia* spp. have also been associated with several common skin disorders including pityriasis versicolor and dandruff as well as more severe inflammatory skin pathologies such as atopic dermatitis (AD) in humans [90] and dermatitis and otitis externa in animals, most frequently in dogs [91]. The mechanism by which *Malassezia* promotes pathology remains largely unclear. A recent study from our lab established an experimental model to study the host response to *Malassezia* in the skin in vivo and thereby revealed a dichotomous role of IL-17 in antifungal immunity as well as in skin inflammation [92]. Topical application of *Malassezia* spp. onto murine skin resulted in rapid and selective induction of IL-17 in the skin by γδ T cells, αβ T cells and to a lesser extent ILCs. In addition, infected animals mounted an adaptive immune response to the fungus in the skin-draining lymph nodes characterized by IL-17- and IL-22-producing Th17 cells, without concomitant Th1 or Th2 priming. Reminiscent of the IL-17 response induced during acute experimental OPC [50], skin-resident Langerhans cells orchestrated the IL-17 response during *Malassezia* skin infection by providing IL-23 [92]. Importantly, IL-17 deficiency resulted in impaired fungal control [92]. Under AD-like conditions, which were mimicked by barrier disruption of the epidermis, the IL-23/IL-17 response to *Malassezia* spp. promoted skin pathology [92], supporting the hypothesis that the skin commensal yeast exacerbates disease severity in AD. The relevance of these data obtained in mice is supported by the observation that the frequency of *Malassezia*-specific Th17 cells was increased in the blood of AD patients compared to healthy individuals [92,93]. Together, these studies provide ample evidence for a role of IL-17-mediated immunity against fungi beyond *C. albicans* that confers host protection, but may also promote immunopathology under certain conditions.

## 5. Concluding Remarks

IL-17 emerged as a key player in antifungal immunity in barrier tissues and many labs have contributed in recent years to the current understanding of the cellular and molecular mechanisms of IL-17 regulation by fungi and effector functions against fungi. The relevance of IL-17 in controlling fungal growth is most prominent for *C. albicans* as evidenced by the predominant occurrence of CMC in patients with genetic defects in the IL-17 pathway. Numerous indications that IL-17 contributes to protection from fungi other than *C. albicans* have been obtained from studies with experimental models of infection. However, it remains less clear to what extent IL-17 is essential for controlling these fungi in humans. This may in part be due to compensatory mechanisms of protection that gain importance if the IL-17 pathway is impaired. The relevance of IL-17 for fungal control may also have been off the grid for fungi, which do not cause symptoms of classical infectious disease if uncontrolled. 

*C. albicans* occupies a unique position as the major inducer of human Th17 responses in healthy individuals [94]. Remarkably, Th17 cells directed against other fungi are induced by cross-reactivity to *C. albicans* [94]. Such cross-reactive Th17 cells also include those with pathogenic potential, e.g., the potential to drive airway inflammation during allergic bronchopulmonary aspergillosis [94]. This notion is supported by a recent study in mice where Th17 responses directed against intestinal colonization with *C. albicans* exacerbate susceptibility to allergic airway inflammation in an experimental model of asthma [95]. Also, Th17 cells directed against other fungi such as *Malassezia* bear the potential to promote immunopathology [92]. Therefore, type 17 immunity directed against commensal fungi provides protection beyond the fungus against which it is originally primed. However, it also acts as a potential risk factor for inflammatory diseases. Understanding the delicate balance between IL-17-mediated protection and pathology in more detail is essential for all aspirations to therapeutically modulate the IL-17 pathway.
